# Biomarkers for sepsis: more than just fever and leukocytosis—a narrative review

**DOI:** 10.1186/s13054-021-03862-5

**Published:** 2022-01-06

**Authors:** Tatiana Barichello, Jaqueline S. Generoso, Mervyn Singer, Felipe Dal-Pizzol

**Affiliations:** 1 Laboratory of Experimental Pathophysiology, Graduate Program in Health Sciences, University of Southern Santa Catarina (UNESC), Criciúma, SC Brazil; 2 Faillace Department of Psychiatry and Behavioral Sciences, McGovern Medical School, The University of Texas Health Science Center at Houston (UTHealth), Houston, TX 77054 USA; 3 Bloomsbury Institute of Intensive Care Medicine, Division of Medicine, University College London, London, UK

**Keywords:** Biomarker, Systemic inflammatory response, Sepsis, Septic shock, Sepsis-associated encephalopathy

## Abstract

A biomarker describes a measurable indicator of a patient's clinical condition that can be measured accurately and reproducibly. Biomarkers offer utility for diagnosis, prognosis, early disease recognition, risk stratification, appropriate treatment (theranostics), and trial enrichment for patients with sepsis or suspected sepsis. In this narrative review, we aim to answer the question, "Do biomarkers in patients with sepsis or septic shock predict mortality, multiple organ dysfunction syndrome (MODS), or organ dysfunction?" We also discuss the role of pro- and anti-inflammatory biomarkers and biomarkers associated with intestinal permeability, endothelial injury, organ dysfunction, blood–brain barrier (BBB) breakdown, brain injury, and short and long-term mortality. For sepsis, a range of biomarkers is identified, including fluid phase pattern recognition molecules (PRMs), complement system, cytokines, chemokines, damage-associated molecular patterns (DAMPs), non-coding RNAs, miRNAs, cell membrane receptors, cell proteins, metabolites, and soluble receptors. We also provide an overview of immune response biomarkers that can help identify or differentiate between systemic inflammatory response syndrome (SIRS), sepsis, septic shock, and sepsis-associated encephalopathy. However, significant work is needed to identify the optimal combinations of biomarkers that can augment diagnosis, treatment, and good patient outcomes.

## Introduction

A biomarker describes a measurable indicator of biological status in normal and pathogenic processes. It may be helpful as a theranostic for identifying suitable patients for therapeutic intervention and titrating the degree and/or duration of intervention. A biomarker should be accurate and reproducible. In the ideal scenario, the biomarker (or combination of biomarkers) should offer both high specificity and sensitivity for diagnosing a condition, but either alone may be adequate as a 'rule-in' or 'rule-out' test.

Sepsis represents a dysregulated immune response to infection that leads to organ dysfunction [1]. Host response biomarkers play a critical role in diagnosis, early recognition of organ dysfunction, risk stratification, prognostication, and patient management, including antibiotic stewardship. Biomarkers may also be helpful for trial enrichment to identify suitable patients and/or risk categorization for an intervention. A wide range of biomarkers, measured by a host of different technologies, are being investigated to discriminate a systemic inflammatory response syndrome (SIRS) rapidly, which is an excessive defensive body's response to a harmful stressor (for example, infection, trauma, surgery, acute inflammation, ischemia or reperfusion, or cancer) [2] or early identification of infection-triggered organ dysfunction (sepsis). Also, the quick sepsis related organ failure assessment (qSOFA) is intended to raise suspicion of sepsis and encourage additional action; although, qSOFA is not a substitute for SIRS [3]. These biomarkers include measurement of acute-phase proteins, cytokines, chemokines, damage-associated molecular patterns (DAMPs), endothelial cell markers, leukocyte surface markers, non-coding RNAs, miRNA, and soluble receptors, as well as metabolites and alterations in gene expression (transcriptomics). Biomarkers may help stratify septic patients into biological phenotypes, for example, hyperinflammatory versus immunosuppressive. Biomarkers can also be used to identify gut permeability, blood–brain barrier (BBB) permeability, probability of hospital readmission, and longer-term outcomes [4, 5].

The causative pathogen replicates and releases its constituents such as endo- and exotoxins, and DNA. These constituents are designated pathogen-associated molecular patterns (PAMPs) [6, 7]. PAMPs are recognized by both pattern-recognition receptors (PRRs) and non-PRRs, which are essential components of the immune system [8, 9]. PRRs include several families, including Toll-like receptors (TLRs), nucleotide-binding oligomerization domain-like receptors (NOD)-like receptors (NLRs), a retinoic acid-inducible gene I (RIG-I)-like receptors (RLRs), C-type lectin receptors (CLRs), and intracellular DNA-sensing molecules. Non-PRRs include receptors for advanced glycation end products (RAGE), triggering receptors expressed on myeloid cells (TREM), and G-protein-coupled receptors (GPCRs) [10]. The sensing of PAMPs by immune cell receptors triggers a cascade of signaling pathways that activate multiple transcription factors to promote the production and release of pro- and anti-inflammatory mediators such as acute-phase proteins, cytokines, chemokines, as well as antimicrobial peptides, which are needed to eliminate the invading pathogen [11].

The host immune response and pathogen virulence factors will both trigger cell injury and/or induce cell stress. These results in the release of endogenous molecules (DAMPs), exacerbating the inflammatory response. DAMPs are recognized by the same immune receptors that recognize PAMPs [12, 13]. Many DAMPs have been identified, with some currently used as inflammatory biomarkers. Examples include proteins and cellular molecules related to nucleic acids, such as heat shock proteins (HSPs), the high mobility group box 1 (HMGB-1), and members of the S100 family [12, 14, 15]. The immune response may induce vascular endothelial damage disrupting tight junctions (T.J.), increasing gut permeability, and potentially facilitating translocation of pathogens and/or their PAMPs from the gut to the bloodstream and lymphatics, thereby amplifying the systemic inflammatory response [16]. In addition, an increase of BBB permeability allows circulating immune cells to enter the brain, triggering or exacerbating glial cell activation [17]. These events could trigger an intense and excessive host response activating coagulation and fibrinolytic systems, activating or suppressing hormonal, bioenergetic, and metabolic pathways, and inducing macro- and microcirculatory changes with a net result of multiple organ dysfunction. In the past few decades, researchers have studied each inflammatory response stage during SIRS, sepsis, and septic shock, metabolites associated with inflammatory cascades, and cellular components that could be used as biomarkers. These biomarkers could help identify endothelial damage, intestinal permeability, organ failure, BBB breakdown and predict rehospitalization, short- and long-term mortality, and cognitive consequences in survivors [18].

For this narrative review, we addressed the question, "Do biomarkers in patients with sepsis or septic shock predict mortality, MODS, or organ dysfunction?" Studies were identified by searching PubMed/MEDLINE (National Library of Medicine) databases for peer-reviewed journal articles published until October 2021. The abovementioned databases were searched with the following combinations of keywords: ("inflammatory response syndrome" OR "SIRS" OR "sepsis" OR "septic shock" OR "sepsis-associated encephalopathy" OR "SAE") AND ("markers" OR "biomarkers" OR "biological markers" OR "biological measures" OR "molecular predictor"). We omitted review articles, in vitro studies, and animal studies.

## The humoral innate immune response, cytokines, and chemokines

The humoral innate immune response consists of multiple components, including fluid phase pattern recognition molecules (PRMs) and the complement system. PRMs include C-reactive protein (CRP), serum amyloid P component (SAP), and pentraxin 3 (PTX-3) [19]. The rise in CRP level is primarily induced by interleukin (IL)-6 and IL-1β acting on the gene responsible for CRP transcription during the acute phase of an inflammatory process. CRP is a pentameric acute-phase reactant protein whose conformation facilitates the ability to trigger complement activation and activate platelets, monocytes, and endothelial cells [20]. Furthermore, CRP is one of the most widely used and investigated biomarkers [21]. A prospective multicenter cohort study followed 483 adult patients who survived hospitalization for sepsis for up to one year. IL-6, high-sensitivity C reactive protein (hs-CRP), soluble programmed death-ligand 1 (sPD-L1), E-selectin, and intercellular adhesion molecule 1 (ICAM-1) were evaluated at five-time points during and after hospitalization. A comparison was made between a phenotype with hyperinflammation (high levels of IL-6 and hs-CRP) and a phenotype of immunosuppression (high sPD-L1 levels). Compared with a normal phenotype, both hyperinflammation and immunosuppression phenotypes had higher 6-month hospital readmission rates and 1-year mortality rates, both all-cause and attributable to cardiovascular or cancer [22].

Pentraxin (PTX-3) is secreted by macrophages, dendritic cells, macrophages, fibroblasts, mesangial cells, and glial cells under pathogen or inflammatory stimuli [19]. Plasma PTX-3 was assessed on days 1, 2, and 7 in 958 patients with sepsis or septic shock included in the Albumin Italian Outcome Sepsis (ALBIOS) study. The researchers assessed a possible association between PTX-3 levels and clinical severity, organ dysfunction, treatment, and mortality within 90 days. PTX-3 levels were elevated at the onset of sepsis and increased with illness severity and the number of organ dysfunctions. PTX-3 levels decreased between days 1 to 7, but this was less prominent in patients with septic shock [23]. In a prospective observational analysis, PTX-3, IL-6, procalcitonin (PCT), and lactate combined showed excellent performance in predicting 28-day all-cause mortality among patients diagnosed with sepsis or septic shock and superior to the Sequential Organ Failure Assessment (SOFA) score [24].

In a prospective pilot study of markers of complement activation in sepsis, higher C4d (3.5-fold), factor Bb (6.1-fold), C3 (0.8-fold), C3a (11.6-fold), and C5a (1.8-fold) levels were seen compared with healthy volunteer controls [25]. In another study of 49 sepsis patients, 34 developed disseminated intravascular coagulation (DIC), and eight died. Patients with DIC had lower C3 levels and higher SC5b-9 levels. On stratifying by SC5b-9 quartile (ng/mL: low: < 260, moderate: 260–342, high: 343–501, highest: > 501), coagulation parameters were most deranged in the highest quartile with prolonged thrombocytopenia and higher mortality (33%) [26].

The activation of PRRs culminates in the stimulation of transcription factors resulting in the expression and secretion of proinflammatory cytokines, including tumor necrosis factor-alpha (TNF-α), IL-1-β, IL-6, and interferons (IFNs). These inflammatory mediators are required for host defense against pathogens and activation of the adaptive immune response. A retrospective study evaluated a broad panel of cytokines and found IL-1β, IL-6, IL-8, MCP-1, IL-10, and plasminogen activator inhibitor 1 (PAI-1) levels were increased in the acute phase of sepsis in both critically and non-critically ill patients. In addition, levels of IL-10 (days 1, 2, and 4), IL-6 and PAI-1 (days 2 and 4), and IL-8 (day 4) increased in critically ill patients compared to non-critically ill [27]. In summary, hs-CRP, IL-6, and PAI-1 circulatory levels may have utility in stratifying a hyperinflammatory patient phenotype.

## DAMPs

DAMPs are endogenous danger molecules released from damaged or stressed cells. These molecules activate the innate immune system through interaction with PRRs. DAMPs contribute to the host defense but can also promote pathological inflammatory responses. Calprotectin, a protein found in the cytosol of neutrophils and macrophages, is released under cell stress or damage. In a mixed population study, plasma calprotectin levels were higher in sepsis than in trauma patients and other medical conditions. Calprotectin levels were higher in patients who did not survive for 30 days. Plasma PCT did not differ between the groups or as a prognosticator of the outcome. Receiver operating characteristic (ROC) analysis, used as a sepsis biomarker, had a higher area under the curve (AUC) value for calprotectin (AUC: 0.79) compared to PCT (AUC: 0.49) [28].

A prospective study evaluated IL-6, HMGB-1, and neutrophil gelatinase-associated lipocalin (NGAL) in 14 septic patients and 16 patients without sepsis admitted to the ICU. In patients with sepsis, IL-6 decreased levels were associated with ICU survival; NGAL levels rose in non-survivors, while HMGB-1 levels were unchanged in both survivors and non-survivors regardless of complications [29].

## Endothelial cells and BBB markers

The first step in endothelial and BBB injury is the breakdown and destruction of proteins followed by release into the bloodstream. These proteins or peptides can be evaluated as a marker of endothelial cells and BBB integrity [30]. Plasma levels of occludin (OCLN), claudin-5 (CLDN-5), zonula-occludens 1 (ZO-1), PCT, and lactate were assessed in 51 septic patients. OCLN and ZO-1 were elevated with disease severity and positively correlated with the Acute Physiology and Chronic Health Evaluation II (APACHE-II) and SOFA scores and lactate levels. The predictive value for in-hospital mortality of ZO-1 was comparable to that of lactate levels, APACHE-II, and SOFA scores but superior to OCLN and PCT [31]. In a case series of brain autopsies from adults who died from sepsis, 38% had no OCLN expression in the endothelium of cerebral microvessels. BBB damage was associated with higher maximum SOFA scores and PCT levels > 10 μg/L. BBB damage in the cerebellum was more common with CRP values > 100 mg/L [32]. Soluble fms-like tyrosine kinase 1 (sFlt-1), soluble E-selectin (sE-selectin), soluble intercellular adhesion molecule 1 (sICAM-1), soluble vascular cell adhesion molecule 1 (sVCAM-1), and PAI-1 were evaluated in another studies. All these evaluated endothelial biomarkers were associated with sepsis severity. sFlt-1 had the strongest association with the SOFA score, while sFlt-1 and PAI-1 had the highest area under the operating receiver characteristic curve for mortality [33].

Syndecan-1 is a structural component of the endothelium. Antithrombin, PAI-1, syndecan-1, VCAM-1, E-selectin, IL-1β, IL-6, IL-8, HMGB-1, and histone-H3 were increased in septic patients compared with healthy controls. Non-survivors had a higher syndecan-1 level compared with survivors. On day one, an association was seen between syndecan-1 levels and APACHE-II, SOFA, DIC scores, hemostatic markers, IL-1β, IL-8, and PAI-1. Day 1 syndecan-1 levels were also significantly higher in patients with DIC and had reliable discriminative power to predict both DIC development and subsequent mortality [34].

The serum biomarker, calcium-binding protein B (S100B), reflects BBB disruption, glial cell injury, and activation. S100B is used to evaluate brain injury severity and predict outcomes from stroke, traumatic brain injury, encephalopathy, and delirium [35]. A prospective cohort study demonstrated that day three values for predicting 180-day mortality were superior to day one (0.731 vs. 0.611) [36]. Patients with sepsis-associated encephalopathy also had elevated levels. In another observational study of 22 patients with septic shock, delirium was present in ten. The odds ratio for the risk of developing delirium with an S100B > 0.15 μg/L was 18.0. Patients with delirium had higher plasma levels of IL-6. S100B and IL-6 levels were positively correlated [37]. S100B, PAI-1, angiopoietin (Ang)-2, ZO-1, and OCLN are the main biomarkers currently used to evaluate the vascular injury and BBB permeability.

## Gut permeability markers

Critically ill patients show an increase in gut permeability, which may trigger a systemic inflammatory response syndrome and multiple organ dysfunction syndromes (MODS) [38]. Plasma zonulin levels were higher in sepsis patients compared to a post-surgical control group or healthy volunteers [39]. In another study, serum levels of intestinal fatty acid-binding protein (I-FABP) were higher in patients with sepsis and higher still in those with septic shock. Serum D-lactic acid levels were also elevated with sepsis but did not differentiate severity. Neither I-FABP nor D-lactic acid could prognosticate [40].

## Non-coding RNAs and miRNA

A non-coding RNA (ncRNA) is an RNA molecule transcribed from DNA but not translated into proteins. A microRNA is a small non-coding RNA molecule that functions in RNA silencing and post-transcriptional gene expression regulation. ncRNAs and mRNAs are being studied as sepsis biomarkers. For example, long non-coding metastasis-associated lung adenocarcinoma transcript 1 (lnc-MALAT1) and micro RNA (miR)-125a were increased in sepsis patients compared with healthy controls and positively correlated with APACHE-II score, SOFA score, serum creatinine, CRP, TNF-α, IL-1β, IL-6, and IL-8. The lnc-MALAT1/miR-125a axis was also a predictor of increased 28-day mortality risk [41]. In another study lnc-MALAT1 expression was increased in acute respiratory distress syndrome (ARDS) patients compared to non-ARDS patients (AUROC: 0.674). Nonsurvivors compared to survivors (AUROC: 0.651) and positively correlated with APACHE-II and SOFA scores, CRP, PCT, TNF-α, IL-1β, IL-6, and IL-17 [42]. Long non-coding RNA maternally expressed gene 3 (lnc-MEG3), and the lnc-MEG3/miR-21 axis were increased, while miR-21 expression was decreased in sepsis patients compared with healthy controls. lnc-MEG3 (AUROC: 0.887) and the lnc-MEG3/miR-21 ratio (AUROC: 0.934) had high values for predicting elevated sepsis risk, while miR-21 (AUROC: 0.801) gave excellent predictive value for a reduced sepsis risk [43]. A further study showed miR-125a and miR-125b expressions were elevated in sepsis patients compared with healthy controls and were predictive of sepsis risk—miR-125a (AUROC: 0.749) and miR-125b (AUROC: 0.839). No correlation was seen between miR-125a and CRP, TNF-α, IL-6, IL-17, and IL-23 in however, miR-125b was positively associated with these cytokines. miR-125a failed to predict 28-day mortality risk (AUROC: 0.588) in sepsis patients, whereas miR-125b was superior (AUROC: 0.699) [44].

## Membrane receptors, cell proteins, and metabolites

Cell surface receptors are receptors incorporated into the plasma membrane of cells and act on cell signaling by receiving or binding to extracellular molecules. After detecting such molecules, the production of metabolites occurs. In one study, the cluster of differentiation (CD)-13, CD14, CD25, CD64, and human leukocyte antigen (HLA-DR) showed acceptable sensitivity and specificity for mortality prediction (CD13 AUROC:0.824; CD64 0.843; and HLA-DR 0.804) while CD14 and CD25 did not predict mortality [45]. nCD64 expression, in a further study, nCD64, PCT, CRP, and SOFA scores were higher in septic patients, with nCD64 having the highest AUC (0.879) for differentiating a positive microbial culture. This was superior to PCT (0.868), SOFA score (0.701), CRP (0.609), and white blood cell (WBC) count. In predicting 28-day mortality, the combination of nCD64 and SOFA score had an AUROC of 0.91 versus 0.882 for the combination of PCT and SOFA [46].

A meta-analysis of 19 studies enrolling 3012 patients evaluated the value of PCT (AUROC 0.84) and presepsin (0.87 AUROC) for diagnosing sepsis. The pooled sensitivities and specificities were 0.80 and 0.75 for PCT and 0.84 and 0.73 for presepsin [47]. In one study, levels of presepsin, PCT, CRP, and WBC were higher in sepsis patients than in a SIRS group with AUROC values of 0.954 (presepsin), 0.874 (PCT), 0.859 (CRP), and 0.723 (WBC). The cut-off of presepsin for discriminating between sepsis and SIRS was 407 pg/ml, with sensitivity and specificity values of 98.6% and 82.6%, respectively [48]. In a study of septic children, TREM-1 levels were higher in septic shock patients [49].

## Hormones and peptide precursors

Adrenomedullin (ADM) is synthesized in different tissues, including the adrenal cortex, kidney, lung, blood vessels, and heart. It has biological properties, including vasodilating, inotropic, diuretic, natriuretic, and bronchodilating. In one study, mid-regional pro adrenomedullin (MR-proADM) was an independent predictor of five different organ failures (respiratory, coagulation, cardiovascular, neurological, and renal), compared to lactate which predicted three (coagulation, cardiovascular and neurological), PCT two (cardiovascular and renal) and CRP (none) [50]. MR-proADM most accurately identified patients with a high likelihood of further disease progression compared to other biomarkers and clinical scores [51]. A total of 1089 individuals with either sepsis (142) or septic shock (977) were included in a randomized controlled study. The MR-proADM level within the first 24 h after sepsis diagnosis was associated with 7-day mortality (AUROC 0.72 and *p* < 0.001) and 90-day mortality (AUROC 0.71 and *p* < 0.001). Patients with declining PCT levels but persistently high MR-proADM levels on day-1 or day-4 had a substantially higher mortality risk of 19.1 (8.0–45.9) and 43.1 (10.1–184.0), respectively [52]. Adult patients hospitalized to ICU had their bioactive-ADM levels measured in this retrospective observational study. This study comprised a total of 1867 patients, 632 septic patients, and 267 septic shock patients. The median bioactive-ADM was 74 pg/mL in sepsis patients, 107 pg/mL in septic shock, and 29 pg/mL in non-septic patients. The association of elevated bioactive-ADM and mortality in sepsis patients and the ICU population resulted in O.R.s of 1.23 and 1.22, respectively [53]. In addition, the MR-proADM is potentially removal by continuous renal replacement therapy (CRRT) [54].

N-terminal (N.T.)-prohormone BNP (NT-proBNP) is a non-active prohormone produced by the heart and released in response to changes in intracardiac pressure. Higher levels of NT-proBNP at 24 h after sepsis onset were associated with lower short physical performance battery (SPPB) scores at 12 months and lower handgrip strength at 6-month and 12-month follow-up. NT-proBNP levels during the acute phase of sepsis may thus be a valuable indicator of a greater risk of long-term impairment in physical function and muscle strength in sepsis survivors [55]. In another study, NT-proBNP levels on admission were higher in-hospital non-survivors (7908 pg/mL) compared with survivors (3479 pg/mL). AUROC curves of admission and 72-h NT-proBNP levels for hospital mortality were 0.631 and 0.648, respectively [56].

PCT is produced in the thyroid's C cells and converted to calcitonin, with no PCT released into the bloodstream. During inflammatory processes, PCT is produced directly by stimulating bacterial components or induced by various inflammatory mediators such as IL-6 and TNF-α. PCT and presepsin had similar performance in predicting positive sepsis results with AUROC values of 0.75 and 0.73, respectively [57]. Another investigation gave AUROC values of 0.87 for PCT and 0.78 for presepsin in predicting bacteremia [58]. Plasma levels of presepsin and PCT were progressively higher in sepsis and septic shock than in non-septic patients. Presepsin was superior to PCT in diagnosing severe community-acquired pneumonia [59], while PCT was marginally superior in another study of septic patients admitted to intensive care [60]. On the other hand, MR-proADM had a better predictive value for septic shock. This study concluded that PCT, MR-proADM, and presepsin are complementary biomarkers that could have utility in the management of septic patients. In an intention-to-treat study comparing PCT versus no PCT guidance, there was no significant difference in 28-day mortality (25.6% PCT versus 28.2% no PCT, *p* = 0.34). The use of procalcitonin did not impact investment decisions as measured by the frequency of therapeutic and diagnostic interventions. [61].

## Neutrophil-related biomarkers

High levels of resistin collected on day 1 of ICU admission were associated with an increased likelihood of developing new organ failure, whereas high myeloperoxidase (MPO) levels on day one were associated with an increased risk of developing incident organ failure for clotting and kidney systems [62].

## Soluble receptors

Soluble trigger receptor expressed in the myeloid cell-1 (sTREM-1) is a TREM family member. This receptor offers excellent potential as a biomarker for infectious diseases as it can be measured in different biological fluids, including serum, pleural fluid, sputum, and urine [63]. However, a meta-analysis of 2418 patients enrolled in 19 studies showed serum sTREM-1 had only moderate accuracy in diagnosing patients with suspected sepsis [63]. Combining sTREM-1 with clinical variables offered more significant mortality discrimination compared to clinical variables alone [64]. In a multicenter prospective cohort study, soluble tumor necrosis factor receptor type 1 (sTNFR1) levels > 8861 pg/ml predicted 30-day mortality [65].

**Fig. 1:**
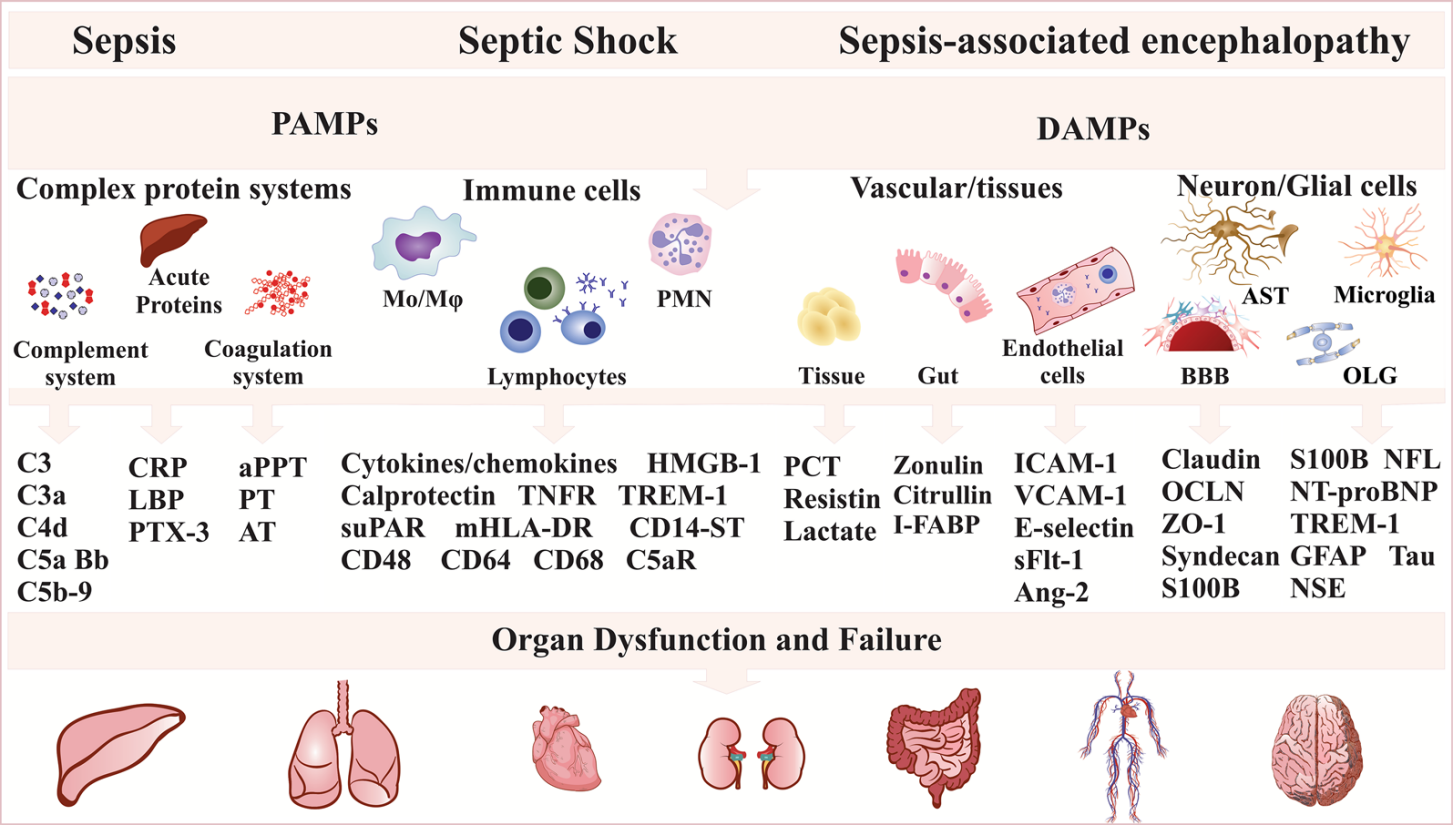
Sepsis, septic shock, and sepsis-associated encephalopathy biomarkers. The infection triggers a cascade of signaling pathways that activate several transcription factors and promote proinflammatory mediators such as acute-phase proteins, cytokines, chemokines, and antimicrobial peptides necessary to eliminate the invading pathogens. The unbalanced host immune response triggers vascular endothelial damage, increasing gut and BBB permeability, culminating in organ dysfunction. Ang-2 (angiopoietin-2), APP (acute phase proteins), aPPT (activated partial thromboplastin), AST (astrocytes), AT (antithrombin), BBB (blood–brain barrier), C5aR (complement component 5a receptor), CD (cluster of differentiation), CD14-ST (soluble subtype of CD14), CRP (C reactive protein), DAMPs (damage-associated molecular patterns), GFAP (glial fibrillary acidic protein), HMGB-1 (high mobility group box 1), ICAM-1 (intercellular adhesion molecule 1), I-FABP (intestinal fatty acid binding protein), LBP (lipopolysaccharide binding protein), mHLA-DR (monocytic human leukocyte antigen DR), Mo (macrophage), NFL (neurofilament light), NSE (neuron specific enolase), NT-proBNP (N-terminal pro-brain natriuretic peptide), OCLN (occludin), OLG (oligodendrocyte), PAMPs (pathogen-associated molecular patterns), PCT (procalcitonin), PMNL (polymorphonuclear leukocytes), PT (prothrombin), PTX-3 (pentraxin-3), S100B (calcium-binding protein B), sFlt-1 (soluble fms-like tyrosine kinase 1), suPAR (soluble form of the urokinase plasminogen activator receptor), TNFR (tumor necrosis factor receptor type), TREM-1 (triggering receptor expressed on myeloid cells 1), VCAM-1 (vascular cell adhesion molecule 1), ZO-1 (zonula-occluden 1)

Patients with sepsis or septic shock displayed higher levels of the soluble form of the urokinase plasminogen activator receptor (suPAR), PCT, and lactate on days 1, 2, 4, and 7 of admission, with lactate and suPAR being the best risk stratifies for suspected infection [66]. Levels of suPAR and PCT levels were higher in sepsis patients than in a SIRS group with AUROC values of 0.89 and 0.82, respectively [67]. Serum sPD-L1 levels were increased in non-survivors compared with survivors with similar prognostic accuracy for 28-day mortality as APACHE-II and SOFA scores [68]. See Tables 1 and 2 for further information, as well as Fig. 1.

**Table 1:** Different roles of the biomarkers in sepsis

Biomarker	Function	References
*Acute-phase proteins*		
CRP, hsCRP	Response to infection and other inflammatory stimuli	[4, 69, 70]
	Predictive for increased 28-day mortality in patients with sepsis	
	Hyperinflammatory phenotype	
Complement	Prognosis of disease severity	[25, 71]
Proteins	C5a can be predictive for DIC	
PTX-3	Discrimination of sepsis and septic shock	[72, 73]
	Diagnosis of sepsis and septic shock during the first week in the ICU	
	Prediction of septic shock	
*Cytokines and chemokines*		
IL-10	Hypoinflammatory phenotype	[22, 71]
MCP-1	It differentiates patients with septic shock from patients with sepsis	[73, 74]
	Mortality prognosis at 30 days and six months	
TNF-α, IL-1β, IL-6	IL-6 all-cause mortality prognosis at 30 days and six months	[27, 74]
	IL-1β and IL-6 acute phase of sepsis	
	It was increased in the hyperinflammatory phenotype	
	Organ dysfunction prognosis	
*DAMPs*		
Calprotectin	PCT to distinguish between patients with sepsis and patients without sepsis in the ICU	[28]
	Predictive for 30-day mortality	
HMGB-1	Worst prognosis and higher 28-day mortality	[75, 76]
*Endothelial cells and BBB markers*		
Syndecan-1	Increase related to sepsis severity	[34]
	Discriminative power for DIC and subsequent mortality	
VLA-3 (a3β1)	Indicative of sepsis	[77, 78]
	Discrimination of sepsis and SIRS	
Ang-1	It stabilizes the endothelium and inhibits vascular leakage by constitutively activating the Tie-2 receptor	[79]
	Ang-2/Ang-1, Ang-1/Tie-2 ratio has a prognosis for 90-day mortality in sepsis and septic shock in the ICU higher than the PCT and SOFA score	
	Independent and effective predictors of SOFA score changes	
Ang-2	It can disrupt microvascular integrity by blocking the Tie-2 receptor, which results in vascular leakage	[73, 79]
	Individuals with septic shock had higher levels of Ang-2 than those with sepsis	
CLDN-5	The absence of CLDN-5 may indicate damage to endothelial cells during sepsis	[31]
OCLN	Increase related to sepsis severity and positive correlation with SOFA scores	[31, 32]
	Predictive of mortality	
	The absence of OCLN in the cerebral microvascular endothelium was related to more severe disease and intense inflammatory response	
PAI-1	Sepsis severity prognosis	[33, 34]
	Predictor of mortality	
	An increase may indicate DIC	
sICAM-1	Sepsis severity prognosis	[33, 79]
	Prognosis of 90-day mortality in patients with sepsis and septic shock in the ICU	
S100B	It is associated with delirium in septic shock	[36, 37, 80]
	Prognosis of severe organ dysfunction	
	Shortest survival in 180 days	
	Diagnosis of sepsis-associated encephalopathy	
E-selectin	Sepsis severity prognosis	[33]
	Predicts mortality	
	Increase related to SOFA and APACHE-II	
sFlt-1	Prognosis of sepsis severity and SOFA score,	[33]
	The prognosis for morbidity and mortality	
sVCAM-1	Prognosis of sepsis severity and 28-day mortality	[33, 79, 81]
	Prognosis of 90-day mortality in patients with severe sepsis and septic shock in the ICU	
	Risk of septic shock	
ZO-1	Prognosis of sepsis severity and correlation with APACHE-II and SOFA scores	[31, 32]
	Predictor of mortality	
	Diagnostic capability for MODS	
*Gut permeability markers*		
Citrulline	The decrease may indicate early acute bowel dysfunction	[82, 83]
I-FABP	Risk of septic shock	[40]
	Indicates early intestinal damage in patients with sepsis and septic shock	
Zonulin	Indicates intestinal permeability during sepsis and SIRS	[39]
D-lactic acid	Indicates early intestinal damage in patients with sepsis and septic shock	[40]
*Non-coding RNAs*		
Lnc-MALAT1	The distinction between septic and non-sepsis patients	[41, 42]
	Positive correlation with APACHE-II	
	Sepsis severity prognosis	
	High risk of ARDS	
	Predictive for high mortality	
	The increase can distinguish ARDS from non-ARDS	
lnc-MEG3	The increase is predictive of sepsis	[43]
	28-day mortality risk	
*miRNA*		
miR-125a, miR-125b	Prognosis of more significant disease severity	[44, 84, 85]
	Distinguishes patients with sepsis from patients without sepsis	
	miR-125b: increased risk of mortality in patients with sepsis	
	miR-125a: risk of sepsis and increased mortality	
*Membrane receptors, cell proteins, and metabolites*		
CD64	Prognosis of disease severity	[46]
	28-day mortality predictor	
	Early diagnosis of infection	
CD68	Prognosis of disease severity	[86]
	Microglial activation	
NFL	Indicates risk and severity of sepsis-associated encephalopathy	[87]
NFH	Indicates risk and severity of sepsis-associated encephalopathy	[87]
NSE	Diagnosis of sepsis-associated encephalopathy	[80, 88]
	30-day mortality risk	
	Risk of delirium	
	Neuronal injury marker in sepsis	
Presepsin	Initial diagnosis and sepsis risk stratification	[48]
	Potential marker to distinguish Gram ( +) and Gram (-) bacterial infection	
TREM-1	Sepsis indicator	[89–91]
	An early distinction between sepsis and SIRS	
	Predictive of septic shock	
*Peptide precursor of the hormone and hormone*		
MR-proADM	Discrimination of survivors and non-survivors	[92]
	Organ dysfunction marker	
PCT	Diagnosis of sepsis	[66, 90]
	Predicts Bacterial Infection	
NT-proBNP	In the acute phase of sepsis it indicates a risk of long-term impairment of physical function and muscle strength	[55]
	Predict mortality risk	[52]
*Neutrophil, cells, and related biomarkers*		
Lactate	Predictive of mortality	[93]
	Risk stratification of patients with suspected infection	
MPO	Increase in patients with DIC	[94, 95]
	Indicates organ dysfunction	
	Mortality predictor at 28 and 90 days	
	Risk of septic shock	
	NET generation	
Resistin	Sepsis indicator	[96, 97]
	Risk of septic shock	
	28-day mortality predictor	
*Soluble receptors*		
sPD-L1	Prognosis of disease severity	[4, 68]
	28-day mortality predictor	
	Indicates immunosuppression	
suPAR	Predictive mortality at 7 and 30 days	[66]
	Risk of patients with suspected infection	
sTNFR-1	Prognosis of 28-day mortality	[81, 98]
	Risk of septic shock	
	Risk of delirium	
*Lipoproteins*		
LDL-C	Protective effect against sepsis	[99]
	The decrease can cause a risk of sepsis and admission to the ICU	
HDL	Low levels: mortality prognosis and adverse clinical outcomes	[100, 101]
	Predictive for MODS	
T-chol	The decrease has a worse prognosis in sepsis	[102]

*Ang-1* angiopoietin-1, *Ang-2* angiopoietin-2, *APACHE-II* acute physiology and chronic health evaluation II, *ARDS* acute respiratory distress syndrome, *CD* cluster of differentiation, *CLDN-5* claudin-5, *CRP* C reactive protein, *DAMPs* damage-associated molecular patterns, *DIC* disseminated intravascular coagulation, *HDL* high-density lipoprotein, *HMGB1* high mobility group box 1, *hsCRP* high-sensitivity C reactive protein, *ICU* intensive care unit, *I-FABP* intestinal fatty acid binding protein, *IL* interleukin, *LDL* low-density lipoprotein, *lnc-MALAT1* long non-coding metastasis-associated lung adenocarcinoma transcript 1, *lnc-MEG3* long non-coding RNA maternally expressed gene 3, *MCP-1* monocyte chemoattractant protein-1, *miR-125a* micro RNA-125a, *miR-125b* micro RNA-125b, *MODS* multiple organ dysfunction syndrome, *MPO* myeloperoxidase, *MR-proADM* mid-regional pro adrenomedullin, *NFL* neurofilament light, *NFH* neurofilament heavy, *NSE* neuron specific enolase, *NT-proBNP* N-terminal pro-brain natriuretic peptide, *OCLN* occludin, *OR* odds ratio, *PAI-1* plasminogen activator inhibitor 1, *PCT* procalcitonin, *PTX-3* pentraxin-3), *S100B* calcium-binding protein B, *sFlt-1* soluble fms-like tyrosine kinase 1, *sICAM-1* soluble intercellular adhesion molecule 1, *SIRS* systemic inflammatory response syndrome, *SOFA* sequential organ failure assessment, *sPD-L1* soluble programmed death ligand 1, *sTNFR1* soluble tumor necrosis factor receptor type 1, *suPAR* soluble form of the urokinase plasminogen activator receptor, *sVCAM-1* soluble vascular cell adhesion molecule 1, *T-chol* total cholesterol, *TNF-α* tumour necrosis factor alpha, *TREM-1* triggering receptor expressed on myeloid cells-1, *VLA-3/a3β1* integrin alpha 3 beta 1, *ZO-1* zonula-occluden 1

**Table 2:** Biomarkers for sepsis, septic shock, and sepsis-associated encephalopathy

Biomarker	Sample	Demographic	Specificity (%)	Sensitivity (%)	Cut-off	*R*^2^	AUC	Clinical relevance	References
Acute-phase proteins									
CRP, hsCRP	Plasma and serum	Sepsis = 483	–	–	15–20 mg/dl	–	–	↑ hsCRP hyperinflammatory phenotype	[22]
		Mean age mean = 60.5	–	–	–	–	–	↑ hsCRP day 1 to 2, 95.8%	
		♂ 54.9%	–	–	–	–	–	↑ hsCRP, 23 patients (25.8%) at 3, 26 patients (30.2%) at 6, and 23 patients (25.6%) at 12 months	
	Plasma	Sepsis = 43	–	–	–	–	0.51, 0.56, and 0.48	CRP, day 1, 3, and 8 to predict 30-day mortality *p* = 0.836, *p* = 0.059, and *p* = 0.819, respectively	[74]
		Septic shock n = 93							
		Age = 26 to 88							
	Plasma	Sepsis = 17	61.54%	52.17%	9 mg/dl	–	0.684	↑ hsCRP sepsis versus control group, *p* = 0.008	[103]
		Control = 19							
		Age = 52.18							
		♂ 63%							
	Serum	Sepsis = 38	100%	88.40%	8.02 mg/l	–	0.98	↑ CRP in septic patients compared to control group, *p* = 0.001	[104]
		Septic shock = 31 Control = 40							
		Age = 37 to 95							
		♂ 57.89%							
	Serum	Sepsis = 27	75.00%	78.00%	7.4 mg/dl	–	0.825	↑ hsCRP sepsis versus control group, *p* = 0.001	[105]
		Septic shock = 23	–	–	–	–	0.751	↑ hsCRP septic shock versus sepsis, *p* = 0.002	
		Control = 20	–	–	–	0.686	–	↑ hs-CRP level versus SOFA, *p* < 0.001	
		Age = 85							
		♂57.89%							
	Blood	Sepsis = 33	90.70%	98.60%	407 pg/ml	-	0.859	↑ CRP in sepsis patients compared in SIRS group, *p* < 0.05	[48]
		Severe sepsis = 24							
		Septic shock = 15							
		SIRS = 23							
		Normal = 20							
		Mean age = 62.1							
	Serum	Sepsis = 119	-	-	-	-	-	↑ CRP and SOFA score in the sepsis group compared to the control group, *p* < 0.05	[46]
		Septic shock = 32						↔ Septic shock group when compared with sepsis group, *p* = 0.086	
		Control = 20	–	–	–	–	–	↔ Diagnosing positive infection culture in patients with sepsis, *p* = 0.071	
			–	–	–	–	0.609		
	Serum	Severe sepsis = 34	–	–	–	–	–	↔ CRP did not differentiate septic shock and severe sepsis	[89]
		Septic shock = 53							
		Age = 2 mo to 16 years							
Complement	Plasma	Sepsis = 20	–	–	–	–	–	↑ Sepsis (C4d 3.5-fold; Factor Bb 6.1-fold; C3 0.8-fold; C3a 11.6-fold; and C5a 1.8-fold) versus control	[25]
Proteins		Control = 10						↑ C5a ↓ SOFA	
		Age = 85	–	–	–	0.18	–		
		♂57.89%					–		
PTX-3	Plasma	Sepsis = 73	–	–	–	0.36	–	↑ PTX-3 versus APACHE-II, and SOFA, *p* = 0.0001	[72]
		Control = 77	–	–	31.4 ng/ml	–	–	Sepsis versus SIRS, *p* > 0.05	
		Septic Shock = 140	–	–	–	–	–	Sepsis versus septic shock, *p* = 0.0001	
		Age = 26 to 88	–	–	–	–	–	↑Sepsis/septic shock versus control, *p* ≤ 0.001	
		♂57.89%	–	–	–	–	0.82 and 0.73	Sepsis and septic shock discrimination on day 1	
	Plasma	Sepsis = 17	–	–	–	–	–	↑ PTX-3 sepsis, septic shock, and post-surgery infection versus control group, *p* < 0.05	[73]
		Septic shock = 26						↑ PTX-3 sepsis shock versus sepsis, *p* < 0.0001	
		Post-surg. Inf. = 33	–	–	–	–	0.798		
		Control = 25							
Cytokines and chemokines									
IL-10	Plasma	Sepsis = 208 Control = 210	–	–	–	–	− 0.161	↑ miR-126 correlated negatively with IL-10, *p* = 0.020	[106]
	Plasma	Sepsis = 309	–	–	–	–	− 0.166	↑ lncRNA ITSN1-2 correlated negatively with IL-10, *p* = 0.003	[107]
		Mean age = 57,3 ± 9,7							
		Control = 330							
		Mean age = 55,8 ± 9,7							
MCP-1	Plasma	Sepsis = 43	–	–	–	–	0.64, 0.51, and 0.51	MCP-1, day 1, 3, and 8 to predict 30-day mortality, *p* = 0.004, *p* = 0.948, and *p* = 0.948, respectively	[74]
		Septic shock n = 93							
		Age = 26 to 88							
	Plasma	Sepsis = 17	–	–	–	–	–	↑ MCP-1 sepsis, septic shock and post-surgery infection versus control group, *p* < 0.05	[73]
		Septic shock = 26						↑ MCP-1 sepsis shock versus sepsis, *p* = 0.0059	
		Post-surg. Inf. = 33	–	–	–	–	0.71		
		Control = 25							
TNF-α, IL-1β, IL-6	Serum	Sepsis = 288	–	–	–	–	–	↑ Sepsis TNF-α, IL-1β, IL-6, and IL-8 compared to the control group, *p* < 0.001	[70]
		Mean age = 58.2 ± 11.2						↑ TNF-α, IL-1β, IL-6, and IL-8 were negatively correlated with surviving sepsis patients, *p* < 0 .001	
		Control = 290	–	–	–	–	− 0.270,		
		Mean age = 56.8 ± 12.1					− 0.310,		
							− 0.254, and		
							− 0.256		
	Plasma	Sepsis = 483	–	–	10^2^ pg/dl	–	–	↑ IL-6, 72 patients (74.2%) at 3 months, 62 (70.5%) at 6 months, and 59 (66.3%) at 12 months	[22]
		Age mean = 60.5							
		♂ 54.9%							
	Serum	Sepsis = 43	–	–	–	–	0.69, 0.70, and 0.68	↑ IL-6, day 1, 3, and 8 to predict 30-day mortality, *p* = 0.0001, *p* = 0.0001, and *p* = 0.012, respectively	[74]
		Septic shock n = 93							
		Age = 26 to 88							
	Serum	Sepsis = 39	–	–	12,704—111,372	–	–	↑ IL-6 septic patients with DIC, *p* = 0.01	[34]
		Control = 15			pg/ml				
		Age ≥ 18 years							
DAMPs									
Calprotectin	Plasma	Sepsis = 77	56%	81%	1.3 mg/l	–	–	↑ Calprotectin, sepsis versus trauma patients, *p* < 0.001	[28]
		Trauma = 32	–	–	–	–	–	↑ Calprotectin at admission was ↑ in non-survivors than in survivors at day 30, *p* < 0.01	
HMGB-1	Serum	Sepsis = 247	–	–	3.6 ng/ml	–	–	↑ HMGB-1 sepsis versus control, *p* < 0.001	[75]
			–	–	–	–	0.51 and 0.53	HMGB-1, day 0 and 3, survivor = non-survivor	
			–	–	–	–	–	HMGB-1 does not have predictive value for organ failure and outcome	
Endothelial cells and BBB markers									
Syndecan-1	Serum	Sepsis = 39	–	–	–	–	–	↑ Syndecan-1 in sepsis versus control, *p* < 0.0001	[34]
		Control = 15	–	–	–	–	–	↑ Syndecan-1 non-survivors on days 1, 2, and 4	
		Age	–	–	–	–	0.54 and 0.59	↑ Syndecan-1 versus DIC on day 1 and 2, *p* = 0.0004 and *p* = 0.0002, subsequently	
		Age ≥ 18 years	–	–	189–1301 ng/ml	–	–	↑ Syndecan-1 in septic patient with DIC, *p* < 0.01	
VLA-3 (a3β1)	Neutrophil	SIRS = 9	–	–	–	–	–	↑ α3β1 (VLA-3, CD49c/CD29) on neutrophils of septic patients, *p* < 0.05	[77]
		Sepsis = 15							
		Control = 7							
		Sepsis = 6	–	–	–	–	–	↑ β1 (CD29), on neutrophils of sepsis patients, *p* < 0.05	[78]
		Control = 5							
Ang-1	Serum	Severe sepsis = 48	–	–	–	–	–	↑ Ang-1 severe sepsis compared with shock septic, *p* < 0.01	[79]
		Septic shock = 54						↓ Ang-1/Tie-2 in non-survivors, *p* < 0.001	
		Age ≥ 18 years	–	–	3.81–16.1 ng/ml	–	–		
	Plasma	SIRS = 943	–	–	–	–	–	↑ Ang-1 was associated with a reduced risk of shock. OR: 0.77	[81]
		Sepsis = 330						↑ Ang-1 was higher in survivor versus non-survivor, *p* < 0.001	
		Shock = 216	–	–	5719 pg/ml	–	–		
		Pneumonia = 169							
		Others = 152							
		Age = 55.1 ± 16.1							
		♂ 63.9%							
Ang-2	Serum	Severe sepsis = 48	–	–	–	–	–	↑ Ang-2 severe sepsis compared with shock septic, *p* < 0.02	[79]
		Septic shock = 54						↓ Ang-2/Ang-1 in non-survivors, *p* < 0.001	
		Age ≥ 18 years	–	–	–	–	–		
	Plasma	SIRS = 943	–	–	–	–	–	↑ Ang-2 was associated with an increased risk of shock, OR: 1.63	[81]
		Sepsis = 330	–	–	42.063 pg/ml	–	–	↑ Ang-2 non-survivor, *p* < 0.001	
		Shock = 216							
		Pneumonia = 169							
		Others = 152							
		Age = 55.1 ± 16.1							
		♂ 63.9%							
CLDN-5	Serum	Sepsis = 11	–	–	–	–	–	↑ CLDN-5 was not associated with MODS and the non-MODS group	[31]
		Severe sepsis = 18	–	–	–	–	0.157 and 0.087	↑ CLDN-5 was not correlated with SOFA or APACHE score, *p* = 0.270, *p* = 0.542	
		Septic shock = 22	–	–	–	–	–	Did not predict mortality	
	Serum	Sepsis = 11	–	–	–	–	–	CLDN-5 was absent from the endothelium	[32]
		Severe sepsis = 18							
		Septic shock = 22							
OCLN	Serum	Sepsis = 11	–	–	–	–	–	↑ OCLN in severe sepsis and septic shock than in sepsis, *p* < 0.05	[31]
		Severe sepsis = 18	–	–	–	–	–	↑ OCLN in non-survivors compared with survivors, *p* < 0.01	
		Septic shock = 22	–	–	–	–	0.337	↑ OCLN positively correlated with SOFA, *p* < 0.016	
			–	–	–	–	0.224	↔ OCLN levels were not correlated with the APACHE-II, *p* < 0.085	
	Brain tissue autopsies	Sepsis = 47	–	–	–	–	–	↓ OCLN, cerebellar endothelium damage, ↑ CRP ≥ 100 mg/l	[32]
			–	–	–	–	–	38% of patients (18/47) had no expression of OCLN in the BMVECs	
			–	–	–	–	–	34% of patients (16/47) had MOFs	
			–	–	–	–	–	74.5% of patients (35/47) had septic shock	
			–	–	–	–	–	The deceased with BBB damage had SOFA scores six versus 14, *p* = 0.04	
PAI-1	Plasma	Sepsis = 63	–	–	–	–	0.85	↑ PAI-1 to predict mortality, *p* < 0.05	[33]
		Severe sepsis = 61	–	–	–	0.45	–	↑ PAI-1 correlated with the SOFA score at 24 h, *p* < 0.0001	
		Septic shock = 42	–	–	–	0.58	–	↑ PAI-1 correlation with APACHE-II score, *p* < 0.0001	
		Age = 60 ± 17	–	–	–	–	–	↑ Severe sepsis ↑ sFlt-1, *p* < 0.0001	
	Serum	Sepsis = 39	–	–	15.5–49.9	–	–	↑ PAI-1 in patients with DIC, *p* = 0.016	[34]
		Control = 15			ng/ml				
		Age ≥ 18 years							
sICAM-1	Plasma	Sepsis = 63	–	–	–	–	–	↑ Severe sepsis ↑ sICAM-1, *p* < 0.001	[33]
		Severe sepsis = 61	–	–	–	0.15	–	↑ sICAM-1 correlated with SOFA score at 24 h, *p* < 0.03	
		Septic shock = 42					–	↑ sICAM-1 correlate with APACHE-II score, *p* < 0.05	
		Age = 60 ± 17	–	–	–	0.17	–		
	Serum	Severe sepsis = 48	–	–	1.297–1787 ng/ml	-	–	↑ sICAM-1 in non-survivors, *p* < 0.001	[79]
		Septic shock = 54	–	–	–	–	–	↑ sICAM-1, predictor of 90 day-mortality, *p* < 0.0001	
		Age ≥ 18 years	–	–	–	–	–	↑ sICAM-1, septic shock compared to severe sepsis *p* < 0.01	
S100B	Serum	Septic shock = 22	–	–	> 0.15 μg/l	–	–	↑ Delirium was present in 10/22 of the patients (45.5%)	[37]
			–	–	–	–	–	OR: 18.0, for risk of developing delirium S-100β > 0.15 μg/l	
			–	–	–	–	0.489	↑ S100 β correlate positively with and IL-6 *p* = 0.021	
	Serum	Sepsis = 104	80.0% and	66.1 and	0.226 and	–	–	↑ S100B cut-of value for day 1 and 3	[36]
		Sepsis-associated encephalopathy = 59	84.44%	69.49%	0.144 μg/l	–	–	↑ S100B in sepsis-associated encephalopathy day 1 to day 3 compared with non- sepsis-associated encephalopathy, *p* < 0.001	
		non- sepsis-associated encephalopathy = 45	–	–	–	–	0.728 and 0.819	↑ S100B on days 1 and 3 to predict sepsis-associated encephalopathy	
			–	–	–	–	0.61	↑ S100B on day 1 to predict 180 day-mortality	
			84.44%	69.49%	0.529 μg/l	–	0.731	↑ S100B on day 3 to predict 180 day-mortality	
			93.33%	50.00%	0.266 μg/l				
	Serum	Sepsis = 21	–	–	–	–	0.082, 0.082,	↑ S100B did not correlate with GCS, EEG pattern, or SOFA scores	[108]
		Age = 68.7					and 0.124		
E-selectin	Plasma	Sepsis = 63	–	–	–	–	0.77	↑ Predict mortality	[33]
		Severe sepsis = 61	–	–	–	–	–	↑ Severe sepsis ↑ sE-selectin, *p* < 0.001	
		Septic shock = 42	–	–	–	0.27	–	↑ sE-selectin correlated with SOFA score at 24 h, *p* < 0.0001	
		Age = 60 ± 17	–	–	–	0.31	–	↑ sE-selectin correlated with APACHE-II score, *p* < 0.0001	
sFlt-1	Plasma	Sepsis = 63	–	–	–	-	0.85	↑ sFlt-1 to predict mortality, *p* < 0.05	[33]
		Severe sepsis = 61	–	–	–	0.36	–	↑ sFlt-1 associated with organ dysfunction	
		Septic shock = 42	–	–	–	0.63	–	↑ sFlt-1 correlation with ↑ IL-6, *p* < 0.0001	
		Age = 60 ± 17	–	–	–	0.6	–	↑ sFlt-1 correlated with SOFA score at 24 h, *p* < 0.0001	
			–	–	–	0.64	–	↑ sFlt-1 correlated with APACHE-II score, *p* < 0.0001	
sVCAM-1	Plasma	Sepsis = 63	–	–	–	–	0.78	↑ Predict mortality	[33]
		Severe sepsis = 61	–	–	–	–	–	↑ Severe sepsis ↑ sVCAM-1, *p* < 0.002	
		Septic shock = 42	–	–	–	0.45	–	↑ sE-selectin correlated with SOFA at 24 h, *p* < 0.0001	
		Age = 60 ± 17	–	–	–	0.38	–	↑ sVCAM-1 correlated with APACHE-II s, *p* < 0.0001	
	Serum	Severe sepsis = 48	–	–	369–467 µg/l	–	–	↑ sVCAM in non-survivors, *p* < 0.001	[79]
		Septic shock = 54	–	–	–	–	–	↑ sVCAM, predictor of 90 day-mortality, *p* < 0.0001	
		Age ≥ 18 years	–	–	–	–	–	↑ sVCAM, septic shock compared to severe sepsis, *p* < 0.01	
	Plasma	SIRS = 943	–	–	–	–	–	↑ s-VCAM was associated with an increased risk of shock. OR: 1.63	[81]
		Sepsis = 330						↑ sVCAM-1 non-survivor, *p* < 0.001	
		Shock = 216	–	–	819 pg/ml	–	–		
		Pneumonia = 169							
		Others = 152							
		Age = 55.1 ± 16.1							
		♂ 63.9%							
ZO-1	Serum	Sepsis = 11	–	–	–	–	–	↑ ZO-1 in severe sepsis and septic shock compared to sepsis, *p* < 0.05	[31]
		Severe sepsis = 18	–	–	–	–	–	↑ ZO-1 in non-survivors compared with survivors, *p* < 0.01	
		Septic shock = 22	–	–	–	–	–	↑ ZO-1 in MODs group	
			–	–	–	–	0.502 and 0.380	↑ ZO-1 was positively correlated with SOFA and APACHE-II scores, *p* < 0.001 and *p* < 0.006	
	Brain tissue autopsies	Sepsis = 47	–	–	–	–	–	ZO-1 is absent from the endothelial cells in the cerebrum and endothelium	[32]
Gut permeability markers									
Citrulline	Plasma	Septic shock = 16	–	–	–	–	–	Citrulline was positively correlated with plasma arginine (r^2^ = 0.85) and glutamine (r^2^ = 0.90) concentrations in both groups, and significantly inversely correlated with CRP (r^2^ = 0.10)	[109]
		(Survivors = 8						↓ Citrulline in patients with digestive bacterial translocation	
		Age = 60 ± 16.5							
		Non-survivor = 8							
		Age = 62.9 ± 18.5	–	–	–	–	–		
	Plasma	Sepsis/ARDS = 44	–	–	–	–	–	↓ Citrulline in all patients	[83]
		Sepsis/NO ARDS = 91	–	–	6 and 10.1 uM	–	–	↓ ARDS compared to the no ARDS group, *p* = 0.002	
		Age = 55 ± 16	–	–	–	–	–	Citrulline levels were associated with ARDS	
I-FABP	Serum	Sepsis = 30	–	–	27.46 and 36.95 μg/l	–	–	↑ I-FABP sepsis and septic shock group, *p* < 0.01	[40]
		Septic shock = 30			–			↑ I-FABP no difference between survivors and non-survivors	
		Control = 20	–	–		–	–		
Zonulin	Plasma	Sepsis = 25	–	–	6.61 ng/ml	–	–	↑ Zonulin sepsis compared to post-surgical and control groups, *p* = 0.008	[39]
		Post-surgical = 18	–	–	–	–	–	No difference between survivors and non-survivors, *p* = 0.305	
		Control = 20	–	–	–	–	0.01, − 0.46, − 0.19, and 0.10	↑ Zonulin, no correlation with CRP, APACHE-II, SAPSII, SOFA, *p* = 0.997, *p* = 0.077, *p* = 0.491, and *p* = 0.671, subsequently	
D-lactic acid	Serum	Sepsis = 30	–	–	15.32 and 27.95 mg/l	–	–	↑ D-lactic acid sepsis and septic shock groups, *p* < 0.01	[40]
		Septic shock = 30	–	–		–	–	↑ D-lactic acid is no different between survivors and non-survivors	
		Control = 20							
Non-coding RNAs									
Lnc-MALAT1	Plasma	Sepsis = 196	–	–	–	–	0.866	↑ Lnc‐MALAT1/miR‐125a axis in sepsis patients	[41]
		Age = 58.2 ± 11.2	–	–	–	–	–	↑ Lnc‐MALAT1 relative expression in sepsis patients	
		Control = 196	–	–	–	–	–	Lnc-MALAT1/miRNA-125a axis discriminates sepsis patients from healthy controls and exhibits a positive association with general disease severity, organ injury, inflammation level, and mortality in sepsis patients	
		Age = 57.1 ± 12.1							
	Plasma	Sepsis = 152	68.50%	65.90%	–	–	0,674 (ARDS	↑ lnc-MALAT1 correlates with raised ARDS risk, disease severity, and increased mortality in septic patients	[42]
		Age = 59.7 ± 11.2	38.30%	88.60%	–	–	0.651	High mortality in sepsis patients	
			–	–	–	–	–	Lnc-MALAT1 expression was positively correlated with inflammatory factor levels (CRP, PCT, TNF-α, IL-1β, IL-6, and IL-17) in septic patients	
	Plasma	Sepsis = 120	–	–	–	–	0.91	↑ lnc-MALAT1 in septic patients, distinguishing patients with sepsis from control	[110]
		Control = 60	–	–	–	–	0.836	Septic shock patients compared to patients without septic shock	
			–	–	–	–	0.886	Non-survivors compared to surviving patients	
			–	–	–	–	–	↑ Lnc-MALAT1 expression was an independent risk factor for sepsis, septic shock, and poor prognosis	
lnc-MEG3	Plasma	Sepsis = 219	–	–	–	–	0,887	↑ lnc‐MEG3 expression predicting elevated sepsis risk	[43]
		Control = 219	–	–	–	–	0.934	lnc-MEG3/miR-21 axis predicting elevated sepsis risk	
		Age = 56.5 ± 10.3	–	–	–	–	0.801	miR-21 was predicting reduced sepsis risk	
			–	–	–	–	0.704	lnc‐MEG3 predicting 28‐day mortality risk	
			–	–	–	–	0.669	lnc‐MEG3/miR‐21 axis predicting 28‐day mortality risk	
			–	–	–	–	–	↑ lnc-MEG3/miR-21 axis, while ↓ miR-21 expression was decreased in sepsis patients	
							-	lnc-MEG3 expression and lnc‐MEG3/miR‐21 axis positively correlated, whereas miR‐21 expression negatively correlated with APACHE-II, SOFA, and inflammatory molecules in sepsis patients	
								↑ lnc‐MEG3 relative expression and lnc‐MEG3/miR‐21 axis in deaths than that in survivor	
miRNA									
miR-125a, miR-125b	Plasma	Sepsis = 120	–	–	–	–	0.557	↔ miR‐125a expression between groups of patients and not differentiate sepsis patients from controls	[84]
		Control = 120	–	–	–	–	0.658	↑ miR-125b in sepsis patients and can distinguish sepsis patients from control healths	
		59.1 ± 12.1	–	–	–	–	–	Positive correlation between miR‐125a and miR‐125b in sepsis patients and controls	
			–	–	–	–	–	miR-125a was not correlated with APACHE-II or SOFA score, while miR-125b was positively associated with both scores	
			–	–	–	–	–	↓ miR-125b in survivors compared with non‐survivors	
			–	–	–	–	–	↑ miR-125b, but not miR‐125a, is correlated with ↑ disease severity, inflammation, and ↑ mortality in sepsis patients	
	Plasma	Sepsis = 126	–	–	–	–	0.817	↓ miR-125a good predictive values for sepsis risk	[85]
		Control = 125	–	–	–	–	0.843	↑ lnc‐ANRIL/miR‐125a axis for sepsis risk	
		Age = 56.6 ± 13	–	–	–	–	0.745	↓ miR‐125a expression in deaths than those in survivors	
			–	–	–	–	0.785	↑ lnc‐ANRIL/miR‐125a differentiating deaths from survivors	
			–	–	–	–	–	lnc-ANRIL/miR-125a axis positively correlated, and miR-125a was negatively associated with disease severity and inflammation in sepsis patients	
	Plasma	Sepsis = 150	–	–	–	–	0.749 and 0.839	↑ miR-125a and miR-125b distinguish sepsis patients from controls	[111]
		Age = 56.9 ± 10.3	–	–	–	–	0.588	miR-125a to predict 28-day mortality risk	
		Control = 150	–	–	–	–	0.699	miR-125b had a potential value in predicting elevated 28-day mortality risk	
		Age = 55.1 ± 11.4	–	–	–	–	–	miR-125a failed to predict the 28-day mortality risk in sepsis patients	
			–	–	–	–	–	1. The predictive value of miR‐125b for sepsis risk	
			–	–	–	–			
								miR‐125a and miR‐125b relative expressions were positively associated with disease severity in sepsis patients	
	Plasma	Sepsis = 196	–	–	–	–	–	↑ lnc‐MALAT1/miR‐125a axis in sepsis patients, *p* < 0.001	[41]
		Age = 58.2 ± 11.2	–	–	–	–	0.931	lnc-MALAT1/miRNA-125a axis discriminates sepsis patients from control	
		Control = 196	–	–	–	–	0.866	lnc-MALAT1 discriminates sepsis patients from control	
		Age = 57.1 ± 12.1							
Membrane receptors, cell proteins, and metabolites									
CD64	Blood	Sepsis = 119	–	–	–	–	–	↑ nCD64 and SOFA score in the sepsis compared to control *p* < 0.05	[46]
		Septic shock = 32	–	–	4.1, 9, and 2.2 MFI	–	–	↑ Sepsis and septic shock compared to control *p* < 0.001	
		Control = 20	–	–	–	–	0.879	nCD64 in bacterial infection	
			–	–	–	–	0.888	↑ AUC of nCD64 combined with SOFA than that of any other parameter alone or in combination	
			–	–	–	–	0.85	CD64 for predicting death	
			–	–	–	–	0.916	Combination of nCD64 and SOFA score	
			–	–	4.1 versus 8.9 MFI	–	–	↑ nCD64 survivors versus non-survivors *p* < 0.001	
	Blood	Sepsis = 20	0.82, 0.67 and 0.67	0.67, 0.76, and 0.76	< 90.40, < 3.01, and < 0.825	–	0.843, 0.824, and 0.804	↑ CD64, ↓CD13, and ↓HLA-DR predict mortality in septic patients	[45]
		Age = 54.35 ± 17.97							
		Control = 20							
		Age = 51.55 ± 13.37							
CD68	Brain	Septic shock = 16	–	–	–	–	–	↑CD68 in the hippocampus (1.5 fold), putamen (2.2 fold), and cerebellum (2.5 fold) in patients with sepsis than control patients	[86]
		Age = 8.9–71.7							
		Control = 15							
		Age = 65.2–87.4							
NFL	CSF and plasma	Sepsis = 20	–	–	1723.4, 1905.2	–	–	Day 1 – sepsis versus control *p* > 0.05	[87]
		Age = 66.7 ± 14.0	–	–	2753.1, 2208.0	–	–	Day 3 – sepsis versus control *p* > 0.05	
		Control = 5	–	–	5309.6, 3701.3 pg/ml	–	–	Day 7 – sepsis versus control *p* > 0.05	
		Age = 61.2 ± 24.7	–	–	–	–	–	↑ NFL in patient septic compared to control from day 1 *p* = 0.0063	
			–	–	–	–	–	↑ NFL patients with sepsis-associated encephalopathy *p* = 0.011	
			–	–	–	–	–	↑ NFL correlated with the severity of sepsis-associated encephalopathy *p* = 0.022	
			–	–		–	–	↑ NFL at CSF in non-survivors compared to survivors *p* = 0.012	
NFH	CSF and plasma	Sepsis = 20	–	–	17.6, 100.3	-	-	Day 1 – sepsis versus control *p* > 0.05	[87]
		Age = 66.7 ± 14	–	–	18.9, 163.1	–	–	Day 3 – sepsis versus control *p* > 0.05	
		Control = 5	–	–	164.3, 519.9	–	–	Day 7 – sepsis versus control *p* = 0.016	
		Age = 61.2 ± 24.7	–	–	ng/ml	–	–	↑ NFH from day 1 in septic patients *p* = 0.043	
NSE	Serum	Sepsis/ sepsis-associated encephalopathy = 48	–	–	24.87 and 15.49 ng/ml	–	–	↑ NSE in sepsis-associated encephalopathy group versus no- sepsis-associated encephalopathy group *p* = 0.003	[80]
		Age = 56 ± 16 Sepsis/non- sepsis-associated encephalopathy = 64			24.15 ng/ml			Diagnostic of sepsis-associated encephalopathy	
		Age = 52 ± 17	82.80%	54.20%	–	–	0.664	↔ NSE, sepsis-survivors versus sepsis-non-survivors *p* = 0.108	
			–	–		–	–		
	Plasma	Sepsis = 124	–	–	> 12.5 ug/l	–	–	23.3%, increased risk of 30-day mortality, *p* = 0.006, and a 29.3% increased risk of delirium *p* = 0.005	[88]
		Mean age = 52–71	–	–	–	–	–	↑ NSE is associated with mortality *p* = 0.003, and delirium in critically ill septic patients *p* < 0.001	
	CSF and plasma	Sepsis/ sepsis-associated encephalopathy = 12	–	–	Eight versus 3.8 ng/ml	–	–	↑ CSF NSE in sepsis group compared to controls *p* < 0.05	[112]
		Control = 21						↔ Plasma NSE sepsis group versus control group	
		Mean age = 67.8 ± 1 2.1	–	–	–	–	–		
Presepsin	Blood	Sepsis = 33	90.70%	98.60%	407 pg/ml	–	0.954	↑ Presepsin in sepsis patients compared to SIRS group *p* < 0.05	[48]
		Severe sepsis = 24						↑ Presepsin and APACHE-II score in severe sepsis group than sepsis group *p* < 0.05	
		Septic shock = 15	–	–	–	–	–	↑ Presepsin and APACHE-II score in septic shock group compared to severe sepsis group *p* < 0.05	
		SIRS = 23							
		Normal = 20	–	–	–	–	–		
		Mean age = 62.1							
TREM-1	Serum	Severe sepsis = 34	–	–	129 pg/ml versus 105 pg/ml	–	–	↑ TREM-1 levels in septic shock compared to severe sepsis	[89]
		Septic shock = 53	–	–	–	–	–	↔ TREM-1 did not differentiate between septic shock and severe sepsis	
		Age = 2 mo to 16 years	56%	60%	116.47 pg/ml	–	0.62	Predict septic shock	
			52%	71%	116.47 pg/ml	–	0.63	Predict mortality	
			–	–	–	–	–	↔ TREM-1 non-survivors versus survivors	
	Serum	SIRS = 38	73.30%	71.10%	≥ 133 pg/ml	–	–	sTREM-1 cut-off for sepsis	[113]
		Sepsis = 52	–	–	–	–	–	↑ sTREM-1 in sepsis group *p* = 0.001	
		Age = 20 to 92	–	–	–	–	–	↑ sTREM-1 in the patients with positive blood culture *p* = 0.002	
	Plasma and leukocytes	Septic shock = 60 Postoperative = 30	100%	98.30%	30.0 pg/ml	–	–	↑ sTREM-1 plasma in septic shock compared to control and postoperative groups *p* < 0.05	[91]
		Control = 30	–	–	–	–	–	↑ sTREM-1 compared with postoperative group *p* < 0.05	
			–	–	–	–	0.955	↑ TREM-1 expression on human monocytes of a septic shock compared to control and postoperative groups *p* < 0.05	
Peptide precursor of the hormone and hormone									
MR-proADM	Plasma	Sepsis/bacterial isolate = 39	78%	74.20%	≥ 1.5	–	0.82	↑ MR-proADM sepsis versus control *p* < 0.0001	[92]
		Sepsis w/bacterial isolate = 23	80%	89.36%	≥ 1.70	–	0.92	↑ MR-proADM septic shock versus control *p* < 0.0001	
		Septic shock = 47	77.40%	59.60%	> 3.00	–	0.7	↑ MR-proADM septic shock versus sepsis *p* < 0.0001	
		Control = 50	–	–	4.37 versus 2.34 nmol/l	–	–	↑ MR-proADM, non-survivor versus survivor *p* < 0.0001	
Bio-ADM		Sepsis = 632	–	–	–		–	Median sepsis patients = 74 pg/mL; septic shock = 107 pg/mL, and 29 pg/mL in non-septic patients	[53]
		Septic shock = 267	–	–	–			Mortality in sepsis patients OR of 1.23	
		Non-septic = 1235	–	–	–		–	↑ Dialysis: OR 1.97 in sepsis patients	
			–	–	70 pg/mL			↑ bio-ADM ↑ Use of vasopressors, OR 1.33	
			–	–	108 pg/mL		–	Survivors and non-survivors in sepsis	
			–	–	–			Youden’s index derived threshold of performed better	
			–	–			–	↑ bio-ADM non-survivors	
							–		
							–		
							–		
PCT	Serum	Sepsis = 59	–	–	–	–	–	↑ PCT *p* < 0.0005	[66]
		Severe sepsis/septic shock = 71	–	–	0.67 versus 3.81	–	–	Survivor versus non-survivor at seven days	
		Mean age = 80	–	–	0.48 versus 1.82 ng/mL	–	–	Survivor versus non-survivor at 30 days	
	Serum	SIRS = 38	65.79%	67.33%	1.57 ng/ml	–	–	PCT cut-off for sepsis	[113]
		Sepsis = 52	–	–	–	–	–	↑ PCT in sepsis group, *p* = 0.01	
		Age = 20 to 92							
	Serum	Sepsis = 79	–	–	–	–	–	↑ PCT concentrations in patients with sepsis and infection	[114]
		Age = newborn to 12						↓ PCT concentrations with antibiotic treatment	
		Control = 21	–	–		–	–		
		Age = newborn to 10							
	Blood	Sepsis = 119	–	–	17.1, 1.8, and 0.04 ng/ml	–	–	↑ PCT septic shock and sepsis compared to the control group *p* < 0.001	[46]
		Septic shock = 32	–	–	1.8 and 9.2 ng/ml	–	–	↑ PCT levels in survivors versus non-survivors *p* > 0.001	
		Control = 20							
	Blood	Sepsis = 33	90.70%	98.60%	407 pg/ml	–	0.874	↑ PCT sepsis patients compared to SIRS group *p* < 0.05	[48]
		Severe sepsis = 24	–	–	–	–	–	↑ PCT and APACHEII score in severe sepsis group compared to sepsis group *p* < 0.05	
		Septic shock = 15							
		SIRS = 23							
		Normal = 20							
		Mean age = 62.1							
	Plasma	Sepsis and shock septic = 1089						↔ There was no statistic difference in the primary outcome regarding PCT-guidance 27.9% versus no PCT-guidance 22.9% to predict mortality *p* = 0.18	[61]
		PCT-guidance n = 279						↔ PCT-guidance versus no PCT-guidance there was no statistic difference in 28-day mortality, 25.6% versus 28.2% *p* = 0.34	
		No PCT-guidance n = 267							
	Serum	Severe sepsis = 34	–	–	129 pg/ml versus 105 pg/ml	–	–	↔ PCT did not differentiate septic shock from severe sepsis	[89]
		Septic shock = 53							
		Age = 2 mo to 16 years							
NT-proBNP	Serum	Sepsis = 60	–	–	1.209 ng/l	–	–	↑ NT-proBNP level at 24 h after sepsis diagnosis	[55]
		Severe sepsis = 89	–	–	–	–	–	↑ NT-proBNP levels at 24 h after sepsis onset were associated with ↓ SPPB scores at 12 months *p* < 0.05, and ↓ handgrip strength at six and 12-month follow-up *p* < 0.001	
		Septic shock = 47							
		Age = 59.1 ± 15.1							
	Plasma	Sepsis = 142			4 (2.6–8.8) versus 8.2 nmol/L (5.2–12.6)		-	↑ NT-proBNP levels in non-survivors compared with survivors *p* < 0.01. ↔ CRP did not change in survivors and non-survivors	[52]
		Septic shock = 947					-	↑ NT-proBNP prediction of 28-day mortality in total population, sepsis group, and shock septic group, respectively	
							0.73, 0.73, and 0.72		
Neutrophil, cells, and related biomarkers									
Lactate	Plasma	Sepsis = 59	–	–	–		–	↑ Lactate *p* < 0.0005	[66]
		Severe sepsis/septic shock = 71	–	–	1.7 versus 3.4		–	Survivor versus non-survivor at seven days	
		Mean age = 80	–	–	1.6 versus 2.2		–	Survivor versus non-survivor at 30 days	
			–	–	mmol/l		0.79 and 0.77	Predictors of mortality at 7 and 30 days *p* = 0.001	
	Serum	Non- sepsis-associated encephalopathy = 2513 Sepsis-associated encephalopathy = 2474	–	–	–	–	–	↑ Lactate predicted 30-day mortality of patients with sepsis-associated encephalopathy, OR: 1.19 *p* < 0.0005	[93]
	Blood	Sepsis = 33	90.70%	98.60%	407 pg/ml	–	0.859 and 0.723	↑ Lactate and APACHE-II score in severe sepsis group compared to sepsis group *p* < 0.05	[48]
		Severe sepsis = 24	–	–	–	–	–	↑ APACHE-II score and lactate in septic shock group when compared with severe sepsis group *p* < 0.05	
		Septic shock = 15							
		SIRS = 23							
		Normal = 20							
		Mean age = 62.1							
	Serum	Severe sepsis = 34	–	–	–	–	–	↔ Lactate did not differentiate septic shock from severe sepsis	[89]
		Septic shock = 53							
		Age = 2 mo to 16 years							
MPO	Plasma	Sepsis = 957	–	–	128.1 ng/ml	–	–	↑ MPO day 1 and progressively decreased until day 7	[94]
			–	–	–	–	–	↑ MPO increase on days on days 1, 2, and 7 in 90-day non-survivors *p* < 0.003, *p* = 0.03, and *p* = 0.001	
		Septic shock = 55	–	–	–	–	–	↑ MPO-DNA and cf-DNA in patients with septic shock on day 1 *p* < 0.01	[95]
		Control = 13	–	–	–	–	–	↑ MPO-DNA on days 3 and 7 of sepsis was associated with 28-day mortality *p* < 0.01	
		Mean age = 68	–	–	–	0.303 and 0.434	–	↑ MPO-DNA on day 3 and 7 positive correlation with SOFA score *p* = 0.04 and *p* = 0.03, subsequently	
		♂ = 71%							
Resistin	Plasma	Sepsis = 957	–	–	192.9 ng/ml	–	–	↑ Resistin on day one and progressively decreased until day 7	[94]
		Mean age = 70	–	–	–	–	–	↑ Resistin increase on days 1, 2, and 7 in 90-day non-survivors *p* < 0.001	
		♂ = 60%							
	Serum	Sepsis = 50	72%, 80%, and 100%	82%, 95%, and 100%	5.2, 6.1, and 7,5 ng/ml	–	–	↑ Resistin levels on day 1, 4, and 7	[115]
		Patient without sepsis = 22	–	–	–	–	0.864, 0.987, and 0.987	↑ Resistin levels on days 1, 4, and 7 were associated with sepsis	
		Control = 25							
		Age ≤ 12							
	Serum	Sepsis = 60	–	–	36.45	–	–	↑ Resistin in sepsis/septic shock groups *p* = 0.001	[96]
		Septic shock = 42	–	–	48.13 versus 31.58	–	–	↑ Resistin levels in non-survivors versus Survivors on day 1 and 7 *p* < 0.001 and *p* < 0.001	
		Control = 102	–	–	46.20 versus 25.22	–	–	↑ Resistin septic shock versus sepsis on day 1 and 3 *p* < 0.001 and *p* < 0.001	
					40.8 versus 33.4				
					37.1 versus 27.4				
					µg/l				
Soluble receptors									
sPD-L1	Serum	Sepsis = 483	–	–	0.16 ng/ml	–	–	↑ sPD-L1 immunosuppression phenotype, ↑ risk of hospital readmission and mortality, OR = 8.26	[22]
		Mean age = 60.5						↑ sPD-L1, 45 (46.4%) at 3 months, 40 (44.9%) at 6 months, and 44 (49.4%) at 12 months	
		♂ 54.9%	–	–	–	–	–	↑ sPD-L1 to predict 28-day mortality ≅ APACHE-II and SOFA scores	
			–	–	–	–	–		
	Serum	Sepsis = 91	–	–	2.09 ng/ml	–	–	↑ sPD-L1 and sPD-1 in septic patients *p* = 0.0001	[68]
		Control = 29	–	–	–	–	–	↑ sPD-L1 increased in non-survivors *p* < 0.05	
			–	–	–	–	0.71	↑ sPD-L1 level to predict 28-day mortality	
suPAR	Serum	Sepsis = 59	–	–	–	–	–	↑ suPAR, *p* < 0.0005	[66]
		Severe sepsis/septic shock = 71	–	–	6.9 versus 9.8	–	–	Survivor versus non-survivor at seven days	
		Mean age = 80	–	–	6.4 versus 9.3	–	–	Survivor versus non-survivor at 30 days	
			–	–	ng/ml		0.72 and 0.77	Predictors of mortality at 7 and 30 days *p* = 0.006	
			–	–	–	–	–	↓ suPAR from day 1 to day seven sepsis and severe sepsis/septic shock *p* < 0.0005	
	Serum	Sepsis = 60	–	–	13	–	–	↑ suPAR in sepsis and septic shock	[96]
		Septic shock = 42	–	–	10.5 versus 14.1	–	–	↑ suPAR in septic shock compared with sepsis on day one but not on day 7 *p* < 0.04 and *p* = 0.68, subsequently	
		Control = 102			11.3 versus 12.9 μg/l				
sTNFR-1	Plasma	SIRS = 943	–	–	7719 versus 18,197	–	–	↑ sTNFR-1 in non-survivor versus survivor, *p* < 0.001	[81]
		Sepsis = 330	–	–	pg/ml	–	–	↑ sTNFR-1 sepsis compared to SIRS *p* < 0.001	
		Shock = 216							
		Pneumonia = 169							
		Others = 152							
		Age = 55.1 ± 16.1							
		♂ 63.9%							
	Plasma	No delirium = 47	–	–	3.843 and 10.250 pg/ml	–	–	↑ sTNFR1 and sTNFR2 delirium cutoff *p* = 0.005	[98]
		Delirium = 31	–	–	–	–	–	↑ sTNFR1 and sTNFR2 in delirium group compared with non-delirium *p* = 0.005, and *p* = 0.003, subsequently	
								OR: 18 to sTNFR1, *p* = 0.004 and OR: 51 to	
								STNFR2, *p* = 0.006	
			–	–	–	–	–		
Lipoproteins									
LDL	Serum	Sepsis = 594	–	–	–	–	–	Risk of sepsis, OR, 0.86, *p* = 0.001and admission to the ICU, OR, 0.85; *p* = 0.008; but not hospital mortality, OR,	[99]
								↓Quartile greater risk of sepsis; OR, 1.48; and admission to the ICU, OR, 1.45, versus highest quartile	
								↔ When comorbidities were considered	
HDL	Serum	Sepsis = 63	–	–	–	–	–	↓ HDL in non-survivors on days 1 to 4	[100]
		Mean age = 72	–	–	–	–	0.84	Predicts mortality within 30 days	
			80%	92%	20 mg/dl	–	–	83% accuracy to predict 30-day overall mortality	
			–	–	–	–	–	HDL < 20 mg/dl increases attributable mortality, risk of prolonged ICU stay, and hospital-acquired infection rate	
	Plasma	Suspected sepsis = 200	0.690	0.716	30.9 mg/dl	–	0,749	MODS predictor	[101]
			0.699	0.857	25,1 mg/dl	–	0,818	Mortality in 28 days	
			–	–	< 25.1 mg/dl	–	–	↑ Mortality, *p* < 0.0001 in 28 days and *p* = 0.0007 in 90 days	
			–	–	–	–	–	74% of patients with HDL < 25.1 mg/dl required ICU compared to 35% above cutoff; development of severe acute renal dysfunction was 47% versus 21%, respectively; multiple organ dysfunction was 60% versus 25%; and mechanical ventilation was 53% versus 21%	
			–	–	–	–	–	↓ HD, the 28-day mortality is more than ten-fold higher (17.6% versus 1.5%) and a mean of 6.2 fewer days without mechanical ventilation and vasopressor support	
T-chol	Serum	Sepsis = 136	–	–	–	–	–	↓ T-chol associated with risk of death in septic patients *p* < 0.05	[102]

*Ang-1* angiopoietin-1, *Ang-2* angiopoietin-2, *APACHE-II* acute physiology and chronic health evaluation II, *ARDS* acute respiratory distress syndrome, *AUC* area under the curve, *BBB* blood–brain barrier, *BMVEC* brain microvascular endothelial cells, *CD* cluster of differentiation, *CLDN-5* claudin-5, *CRP* C reactive protein, *CSF* cerebrospinal fluid, *DAMPs* damage-associated molecular patterns, *DIC* disseminated intravascular coagulation, *EEG* electroencephalography, *GCS* Glasgow coma scale, *HDL* high-density lipoprotein, *HLA-DR* human leukocyte antigen, *HMGB1* high mobility group box 1, *hsCRP* high-sensitivity C reactive protein, *I-FABP* intestinal fatty acid binding protein, *IL* interleukin, *LDL* low-density lipoprotein, *lnc-ANRIL* long non-coding antisense non-coding RNA in the INK4 locus, *lnc-MALAT1* long non-coding metastasis-associated lung adenocarcinoma transcript 1, *lnc-MEG3* long non-coding RNA maternally expressed gene 3, *lncRNA* long non-coding RNA, *MCP-1* monocyte chemoattractant protein-1, *miR-125a* micro RNA-125a, *miR-125b* micro RNA-125b, *MODS* multiple organ dysfunction syndrome, *MOF* multiple organ failure, *MPO* myeloperoxidase, *MR-proADM* mid-regional pro adrenomedullin, *NFL* neurofilament light, *NfH* neurofilament heavy, *NSE* neuron specific enolase, *NT-proBNP* N-terminal pro-brain natriuretic peptide, *OCLN* occludin, *OR* odds ratio, *PAI-1* plasminogen activator inhibitor 1, *PCT* procalcitonin, *PTX-3* pentraxin-3, *RNA* ribonucleic acid, *S100B* calcium-binding protein B, *sE-Selectin* soluble E-selectin, *sFlt-1* soluble fms-like tyrosine kinase 1, *sICAM-1* soluble intercellular adhesion molecule 1, *SIRS* systemic inflammatory response syndrome, *SOFA* sequential organ failure assessment, *sPD-1* soluble programmed death protein 1, *sPD-L1* soluble programmed death ligand 1, *SPPB* short physical performance battery, *sTNFR1* soluble tumor necrosis factor receptor type 1, *sTNFR2* soluble tumor necrosis factor receptor type 2, *sTREM-1* soluble triggering receptor expressed on myeloid cells 1, *suPAR* soluble form of the urokinase plasminogen activator receptor, *sVCAM-1* soluble vascular cell adhesion molecule 1, *T-chol* total cholesterol, *TNF-α* tumour necrosis factor alpha, *TREM-1* triggering receptor expressed on myeloid cells-1, *VLA-3/a3β1* integrin alpha 3 beta 1, *ZO-1* zonula-occluden 1). ↑ increase, ↓ decrease, ↔ no difference

## Conclusion

Despite significant advances in treating septic patients, this disease continues to be associated with high mortality rates and high long-term cognitive dysfunction. Extensive research in the area is being performed to validate biomarkers, facilitate sepsis diagnosis, and allow an early intervention that, although primarily supportive, can reduce the risk of death. Sepsis sometimes shows a hyperinflammatory response pattern and may be followed by an immunosuppressive phase, during which multiple organ dysfunction is present. A biomarker or a panel of biomarkers could be a new avenue to predict, identify, or provide new approaches to treat sepsis.

## Data Availability

Not applicable.
